# A screening and prevention programme serving an ethnically diverse population of women at high risk of developing breast and/or ovarian cancer

**DOI:** 10.3332/ecancer.2008.123

**Published:** 2009-03-16

**Authors:** J Smith, L Baer, S Blank, A Dilawari, K Carapetyan, M Alvear, M Utate, J Curtin, F Muggia

**Affiliations:** 1 Department of Medical Oncology, New York University, USA; 2 Department of Medicine, SUNY, Stony Brook, NY, USA; 3 Department of Gynecology, New York University, USA; 4 Langone Medical Center, New York University, USA

## Abstract

**Introduction:**

We describe a screening and prevention programme primarily targeting under-served minority women at high risk of breast and/or ovarian cancer. Women attending this Bellevue Hospital Center (BHC) Clinic were either self-referred from a variety of special outreach programmes or referred internally by medical professionals caring for relatives or friends. Our objective was to delineate referral sources and preliminary risk-assessment findings in relation to demographic features in this population.

**Methods:**

Following a detailed family and personal history intake and physical examination, each woman on her initial visit is categorized into a low (standard) risk, high-risk or indeterminate-risk group. Women found to be at high risk of developing breast and/or ovarian cancers are referred for further testing, additional screening measures, or participation in chemoprevention trials. All other women are counselled concerning follow-up and lifestyle issues.

**Result:**

Between 2003 and 2007, 171 women for whom complete information was obtained were analysed. Thirty-four of the women were Caucasians (19.8%) and 137 (80.2%) were ethnically diverse minority women. Sixty-two (36.2%) were found to be at high risk with a median age of 42 years. The majority of the high-risk women were referred to the clinic by medical professionals (58%), most of whom were from within the BHC health care system. In fact, one-fourth of the referrals were women who carried a diagnosis of cancer, mostly arising in the breast, and who were concerned with risks to other family members. Trends in genetic testing results indicate fewer mutations among high-risk Asians than among other ethnicities.

**Conclusion:**

Accurate risk assessments and implementation of screening and prevention measures have been challenging during the first few years of operation. Nevertheless, the need for providing consultation from internal referrals and the potential for genetic and psychosocial research in an ethnically diverse population are powerful incentives for continuing to evolve these services.

## Introduction

An estimated 5–10% of all breast cancer cases are attributed to inherited mutations in genes conferring breast cancer susceptibility as high as 80% life-time risk [1]. By their late thirties, BRCA1 mutation carriers have a 2–3% risk of developing ovarian cancer, and that risk rises dramatically with advancing age [2]. Early identification of women at risk of being a carrier of one such mutation is becoming increasingly important: a positive carrier state may lead to intensified screening according to recommended guidelines, or to risk reducing surgeries such as bilateral salpingo-oopherectomy (BSO) and/or mastectomy [3–5]. The timing of risk reducing surgery is influenced by this hereditary risk in conjunction with the reproductive wishes of the patient.

Studies have shown that the prevalence of these mutations vary greatly between populations and ethnicities; for example while certain founder mutations in BRCA1 are reported to be the most common in Ashkenazi Jews [6–8], a single mutation in BRCA2 can be found in the majority of high-risk families in Iceland [9]. Other ethnic minorities remain understudied and little is known about the true prevalence of the documented deleterious mutations and the associated lifetime risk of developing breast and/or ovarian cancer. The reasons for this information disparity are multi-factorial and have been documented in many studies: language barriers, cultural differences, lack of medical insurance and limited access to specialized care being some of the main explanations cited [10–13]. Geographical issues may contribute to the difficulties. Some minorities tend to reside in specific US locales, thus being under-represented in studies originating from other parts of the US. Being part of an academic centre serving a diverse population located in a major metropolitan area has facilitated our reaching out to under-served minorities. From its outset in 2001, the Lynne Cohen High Risk Clinic targeted such minority and under-served populations as the main beneficiaries of its services. The purpose of this report is to evaluate the demographics, risk and referral sources of the women attending the clinic focusing on recent years of operation when criteria for high-risk designation were uniformly applied. In our particular working environment of a major medical facility caring for immigrant populations, one-fourth of all the subjects were women with an established history of cancer, accounting for nearly half of the women that were subsequently identified as high risk. This experience points to an area of major need in caring for women with cancer (i.e. counselling family) as well as a likely strong consideration for subjects seeking cancer screening and prevention (i.e. breast cancer in a first-degree relative).

## Methods and materials

Since 2001, 398 women were registered free of charge in the Lynne Cohen High Risk Clinic as part of a research project designed to identify women at high risk of developing breast and/or ovarian cancer. The resources utilized originated at the NYU Cancer Center and the Lynne Cohen Foundation. Referrals were from BHC various specialties as well as Center for Immigrant Health of NYU. The High Risk Clinic Staff has attended departmental conferences and tumour boards in relevant units such as Internal Medicine, Geriatrics, Gynecology, Gynecology-Oncology, and Medical Oncology. The goals of the clinic, services offered and eligibility criteria were presented to increase awareness and promote referrals.

Collaboration with outreach and community programmes was sought out to help promote access for immigrant and minority women. These included the YWCA, the American Cancer Society Eastern Division, Latina Share, Cancer Care and the Women’s Outreach Network. Relatives of women found at high risk were also encouraged to enrol.

Upon arrival, women go through the following procedure: women meet a bilingual nurse who explains the sequence of the visit and assists in filling out a comprehensive clinical and research questionnaire, available in both English and Spanish. Ethnic and demographic data and an expanded personal and family history (modified from the Gynecologic Oncology Group Protocol 199) are included [14]. Mammography findings and benign breast disease (including number of biopsies and results) and gynaecologic history (age of menarche, age at menopause, age at first and last full-term pregnancies, duration of lactation, use of contraceptives), any hormonal interventions and other past medical history with medications are recorded. An oncologist reviews the data at the clinical encounter, which is an interview and physical examination, including breast and pelvic exams. If applicable (women over 35 with no personal history of breast cancer), the National Cancer Institute’s breast cancer risk assessment tool is used to assess each individual women’s risk. This widely used and validated tool is based on a modified Gail model that factors in ethnicity as white/Caucasian, Hispanic, African-American or Asian (Rockhill *et al* [23]) and is available at http://www.cancer.gov/bcrisktool/. Based on the interview and risk assessment models, the woman’s risk profile is discussed, with recommendations for surveillance and follow-up.

Since 2004, women estimated to be at an increased risk of breast and ovarian cancer based on personal or family history are referred to a dedicated genetic counsellor for a formal consultation in order to asses risk and determine need for genetic testing based on the clinic’s guidelines (Table 1). The information is then entered into a computerized database by a dedicated data manager. Monthly multidisciplinary clinic staff meetings are held to discuss each woman’s risk profile. Based on the completed questionnaire, the history and physical examination, and the genetic consultation if performed, women are stratified to one of three risk groups: low (standard), high and indeterminate.

Women at *low risk* have no established risk factors and are referred for standard follow-up: mammograms every 1–2 years after age 40 and annual visits with a primary care physician. Women at *high risk* are those with genetic risk factors (based on proximal or more distant but extensively affected family members), clinical risk factors, or a known diagnosis on biopsy such as atypical hyperplasia or lobular carcinoma *in situ*. These women are followed up every six months with physical and pelvic examinations and are eligible to undergo genetic testing (Table 1). When not covered by insurance, this testing is carried out utilizing research funds. Positivity for BRCA1 or BRCA2 mutation determines eligibility to certain clinical trials, as well as decision of referral for risk-reducing surgeries. Mammography is recommended to begin ten years prior to diagnosis of the affected relative, with follow-up mammograms every six months.

An *indeterminate risk* is assigned if the information on family history or prior pathology is insufficient or requires confirmation prior to classifying risk level.

## Results

We base our analysis on 171 new referrals seen from 2004 through 2007, with complete data: of the 171 women, 34 were Caucasians (19.8%) and 137 (80.1%) were ethnically diverse minorities (Table 2). Assignation to high risk was made in 62 of the women. The percentage of high-risk designation was higher in Asian women, and Ashkenazi Jewish women attending the clinic (58.8% and 57.1%, respectively), while only 26.9% of the Latina women, the most heavily represented ethnic group, were at high risk.

The majority of women both as a whole and in most ethnic groups were referred to the clinic by medical professionals, most from within the Bellevue health system itself (Table 3). The second most common referral source in minority women was community agencies and outreach programmes. Together these accounted for about a quarter of the referrals. In contrast, these agencies and outreach programmes comprised the most common referral source for Caucasian women.

In the group referred by medical professionals, a common reason in the high-risk cohort was a personal history of cancer. This was the referral reason for 30 of the women in the high-risk group. The breakdown of the specific cancer types is as follows: 23 of these 30 had a diagnosis of pre-menopausal breast cancer and three had ovarian cancer, two of which were serous papillary carcinomas and one of which is a tumour of low malignant potential (LMP). Three women had been diagnosed with other primary cancers, including stomach cancer, endometrial carcinoma and vulvar melanoma. Three women with early onset breast cancer developed bilateral disease, and a fourth was later diagnosed with endometrial cancer (Table 4). In the absence of a personal history of cancer, women were referred to the clinic from a routine screening test site, such as a mammogram or routine gynaecological examination. These referrals were prompted by a family history suggestive of familial clustering of breast and/or ovarian cancer. It is this same history that motivated women to attend the clinic after being informed of its existence by a community agency or an outreach programme.

Twenty per cent of all the women who registered in our clinic, and mostly among the high-risk group, belonged to ten families with two to five members (median 2) being screened; only two families were of Caucasian ethnicity. A diagnosis of breast cancer in first-degree relatives prompted referral in seven (six with one relative, and one with two relatives), whereas a diagnosis of ovarian cancer in a first-degree family member was the major impetus for referral in three families. Mother--daughter referrals outnumbered sister–sister referrals.

Genetic testing for BRCA mutations has been carried out since 2003 and more than half of these since 2007 when special funding was obtained from an institutional grant (see acknowledgement). The ethnicities of all women seen in the clinic from 2003 to 2007 is illustrated in Table 2. Since 2003, 88 women were recommended for genetic testing, 44 had the test performed. Sixteen per cent of the women tested were African-American (including Caribbean and West Indies origin), 27% Latina, 32% Asian and 3% of Eastern European Jewish origins. Among the ten high-risk Asian women seen during that time period, seven were tested and one was a carrier for the BRCA 2 mutation, the other two had variants of unknown significance, BRCA 1 (V447A) and BRCA 2 (V2049M).

## Discussion

Providing minority women risk-guided screening and prevention opportunities remains challenging. While some women may be highly motivated by self-perceived increased risk due to multiple cases of cancer in the family, many barriers exist preventing their enrolment into clinics and trials. Our clinic set out to provide specialized care at no cost thereby eliminating the possible lack of insurance as a major obstacle to care. Language barrier problems were dealt with by the presence of bilingual nurse and patient navigators; however, the difficulty of reaching the target population to inform them of the clinic’s services, remained an issue. Over the course of the first six years, the clinic staff has dedicated much of its activity to educational initiatives for the medical community, and to the formation of bonds with community and outreach programmes. The specific organizations approached were chosen for their work with particular populations and their agenda of promoting health education, screening and prevention. The most highly represented ethnic group, which also comprises the largest group of women at high risk, is the Latina. Although Ashkenazi Jewish and Asian women were referred less frequently to our clinic, the relative percentage of high-risk women was the highest in these groups.

A similar approach of mapping high-risk populations and forming partnerships with trusted community institutions in order to maximize penetration to these populations was described by Barry and Britt, in their paper describing efforts to promote cervical and breast cancer screening in impoverished minority women [19]. While this approach may be fruitful in recruitment of normal risk women, its success as a recruitment tool for women at high risk for development of breast and/or ovarian cancer remains to be determined. Our efforts were directed both at the medical community and at the community outreach programmes. Most women (49.6%) were referred via medical professionals, 28% were referred through outreach organizations and programmes. The percentages were even more clearly divided in the group of women who were at high risk with 58% referred by medical professionals and 24% by outreach sources. A similar breakdown was observed with all ethnic groups, with the exception of Asian women of which 70% were referred by medical entities. The assistance of a dedicated patient navigator fluent in the common Chinese dialects likely resulted in the high recruitment of Asian women from the Bellevue medical clinics. In their paper, describing the establishment of under-served cancer genetics services clinic, Ricker *et al* described a similar dual approach for both in- and outreach [20]. It is important to understand, through models such as our clinic, how effective techniques of communication, outreach and education can lead to targeting the populations at risk. In this context, it is noteworthy that one-fourth of all the subjects were women with an established history of cancer, and that these accounted for nearly half of all women subsequently identified as high risk. This experience points to counselling families as an area of major need in caring for women with cancer, and conversely, to a diagnosis of breast cancer being a major impetus for women to seek screening and prevention advice for their families. The data obtainable from experience in clinics such as ours might lead to different paradigms for determining and encouraging entry into clinical trials, such as those that have been directed to breast cancer prevention but have enrolled mostly women of Caucasian origin [21–23].

## Conclusion

In conclusion, our clinic has been a valuable resource for internal referral of affected patients and family members of patients with breast cancer to discuss their risk. It also relies on external referrals that depend on awareness in communities within BHC catchment area. Classification into risk based on sometimes inadequate family information and challenges in obtaining genetic testing have been our major obstacles. On the other hand, the Lynne Cohen Foundation for Ovarian Cancer Research has sponsored research symposia activities and the formation of a consortium with four institutions including our own. This Lynne Cohen Foundation Consortium includes the MD Anderson Cancer Centers, the University of Alabama, the University of Southern California as well as our own, and the creation of a common registry represents an opportunity for subsequent collaborative research that may include prospective clinical trials.

## Figures and Tables

**Table 1:**
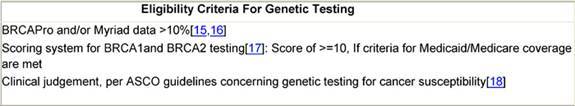
Eligibility criteria for genetic testing

**Table 2:**
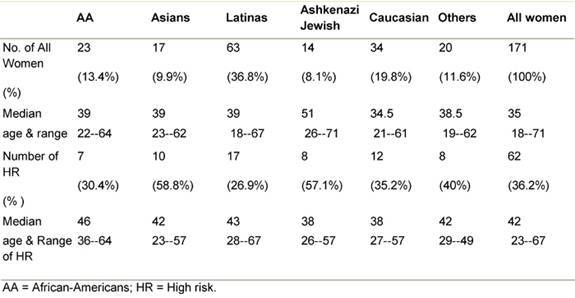
Patients by ethnicity, risk and age

**Table 3:**
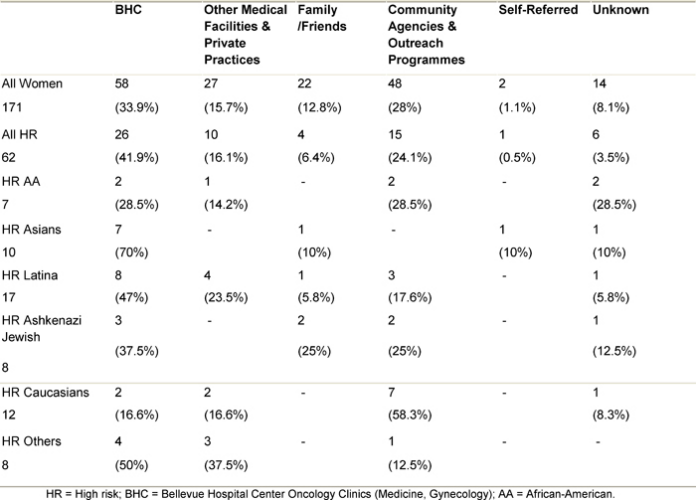
Source of referral to the HR clinic by ethnicity

**Table 4:**
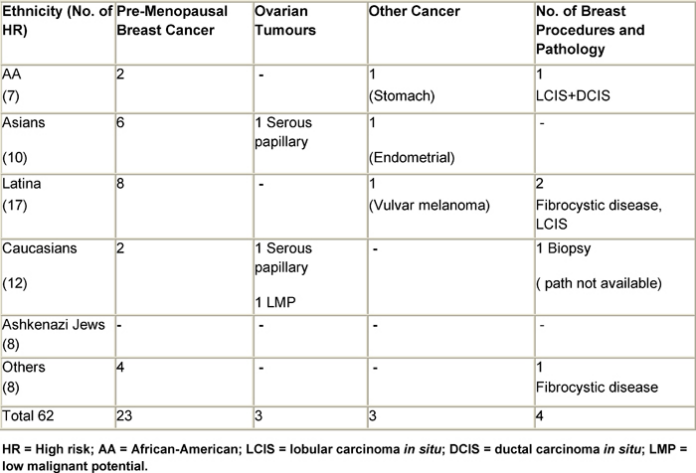
Primary neoplasms and breast procedures in HR women
